# Supplementary material for: Reduction of corpus callosum activity during whisking leads to interhemispheric decorrelation
Source: Nat Commun. 2021 Jul 2;12:4095. doi: 10.1038/s41467-021-24310-6 (PMC8253780; doi:10.1038/s41467-021-24310-6)
Supplement: Supplementary file 1 — Supplementary Information [file 41467_2021_24310_MOESM1_ESM.pdf]

Supplemental Information for:

Reduction of corpus callosum activity during whisking leads to interhemispheric decorrelation

Yael Oran*, Yonatan Katz*, Michael Sokoletsky, Katayun Cohen-Kashi Malina, Ilan Lampl

Supplementary Figure 1:

Correlation between whisker pad movements during free air whisking

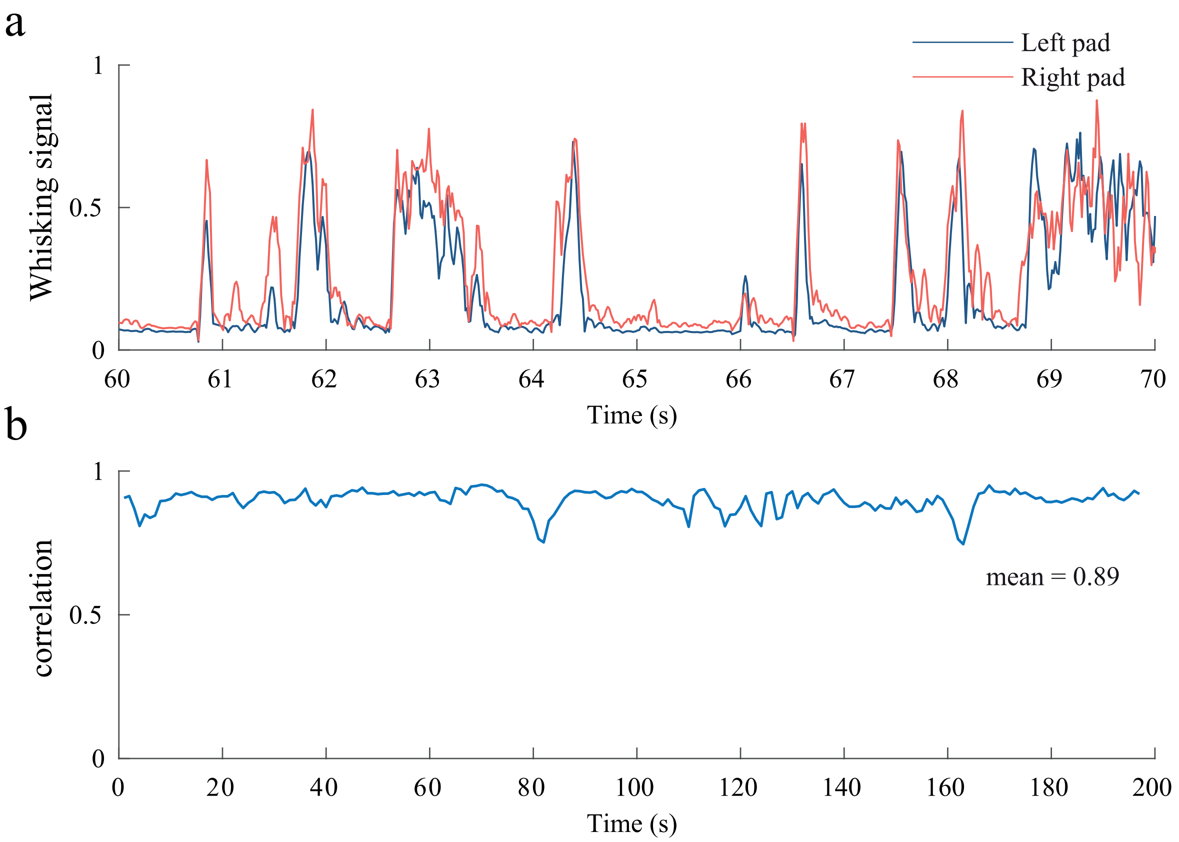

a. Example of whisker pads movement during whisking (blue-Left pad, red-Right pad). **b.** Cross-correlation between the two whisker pads over time.

Supplementary Figure 2:

Interhemispheric cross-correlations during ongoing activity for homotopic and non-homotopic barrels are similar.

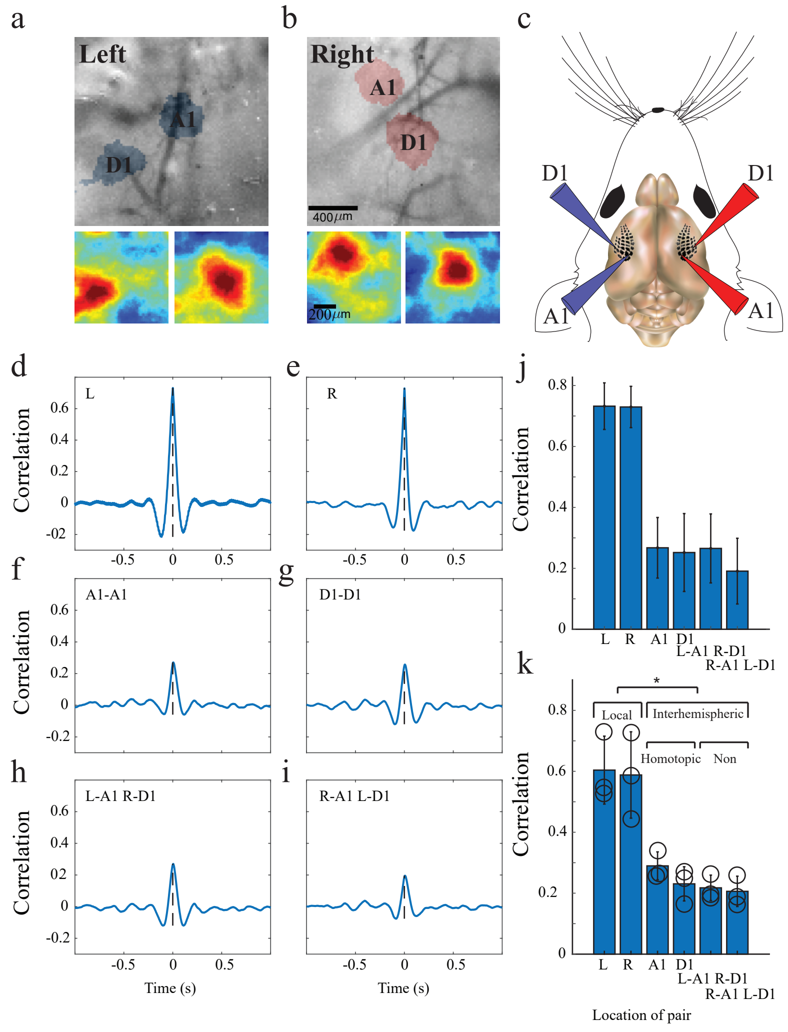

a, b. An example from one animal (out of 3) of intrinsic imaging maps of A1 and D1 whiskers at the left (a) and right (b) hemispheres, on top of blood vessels, the image was taken through a thinned skull (scalebar 400 µm for both panels). Lower panels show intrinsic imaging signal maps produced by stimulation of A1 or D1 whiskers at the contralateral side. scalebar 200 µm for all panels. **c.** Schematic of LFP recordings in awake mice in left A1, D1, and right A1, D1 barrel columns following intrinsic imaging. **d-e.** Cross-correlations of ongoing activity between the LFP signals recorded in the A1 and D1 barrels of the same hemisphere (d - left hemisphere, e-right hemisphere). **f-g.** Interhemispheric cross-correlation between homotopic barrels, recordings were made from A1-A1 columns (f) and D1-D1 columns (g). **h-i.** Interhemispheric cross-correlations between non-homotopic barrels, recordings were made from A1-left hemisphere and D1-right hemisphere (h) and from A1-right hemisphere and D1-left side (i). **j.** Pearson correlation of the example pair LFP recordings (n = 148 trials mean ± SD) in h-i showing high local correlation (L, R). Interhemispheric correlations obtained from homotopic versus non-homotopic barrel columns were similar (i.e., the last four bars). **k.** Population average for all animals (n = 3), Two-way ANOVA and Tukey's HSD post-hoc test for multiple comparisons (mean ± SEM, * $p < 0.05$).

Supplementary Figure 3:

The ratio of quiet wakefulness versus whisking state

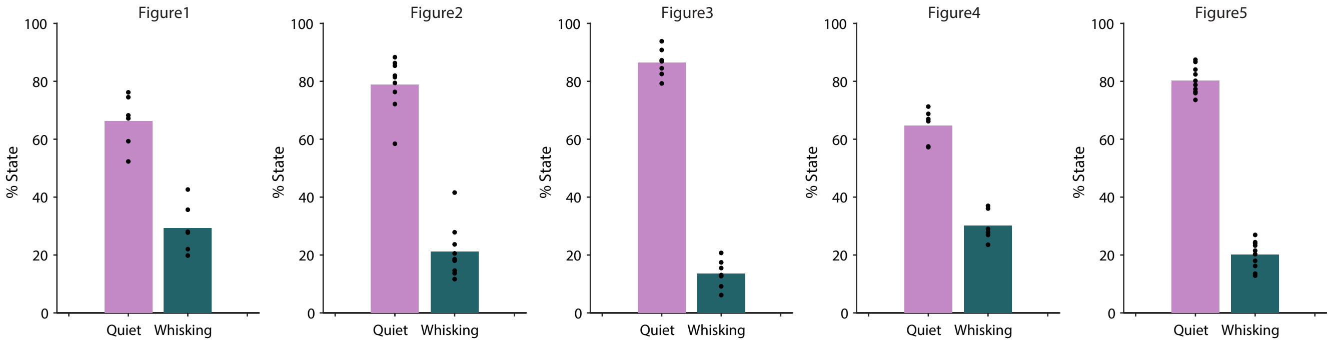

Percentage of time mice spent in quiet wakefulness (pink) vs. Whisking (teal) states at each experiment shown in the main figures of the manuscript. The proportion of time spent in whisking state varied from 14% (Figure 3) to 30% (Figure 4). Data points represent recording sessions.

Supplementary Figure 4:

STA between hemispheres

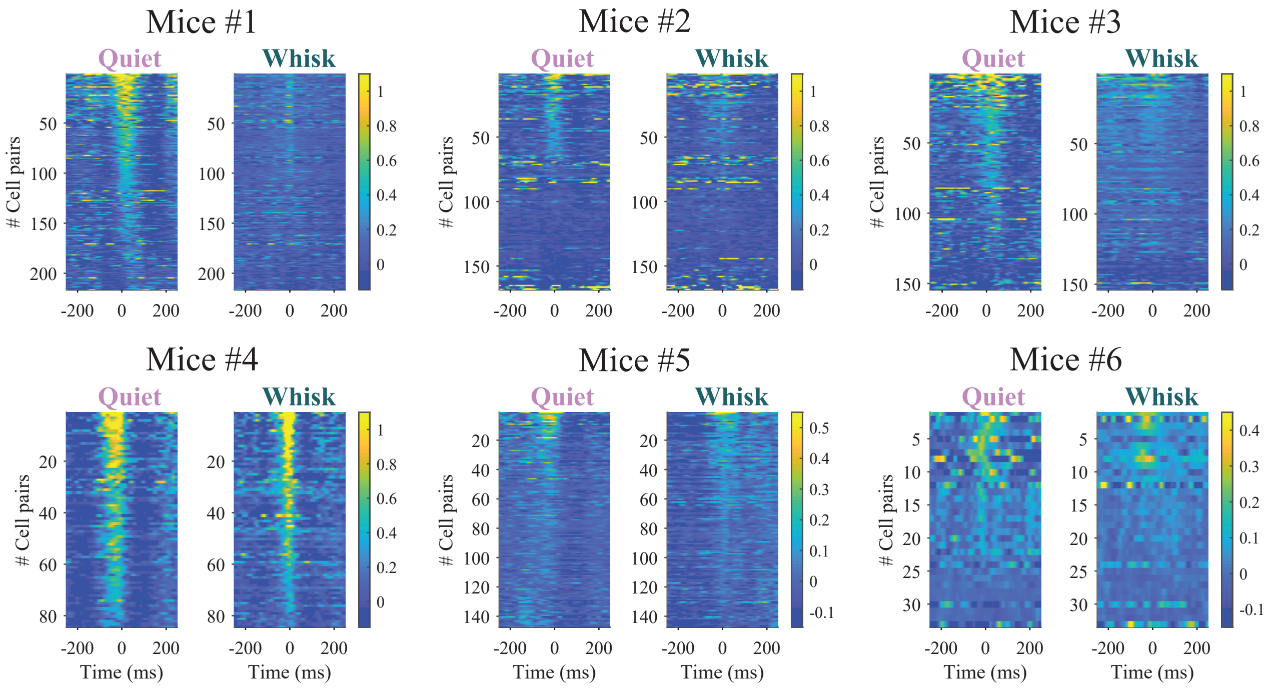

STAs of all cell pairs between hemispheres during quiet wakefulness and whisking states for each animal. Both panels are ordered identically, based on mean peak STAs obtained from both states.

Supplementary Figure 5:

Interhemispheric subthreshold correlations for raw and low pass filtered membrane potential.

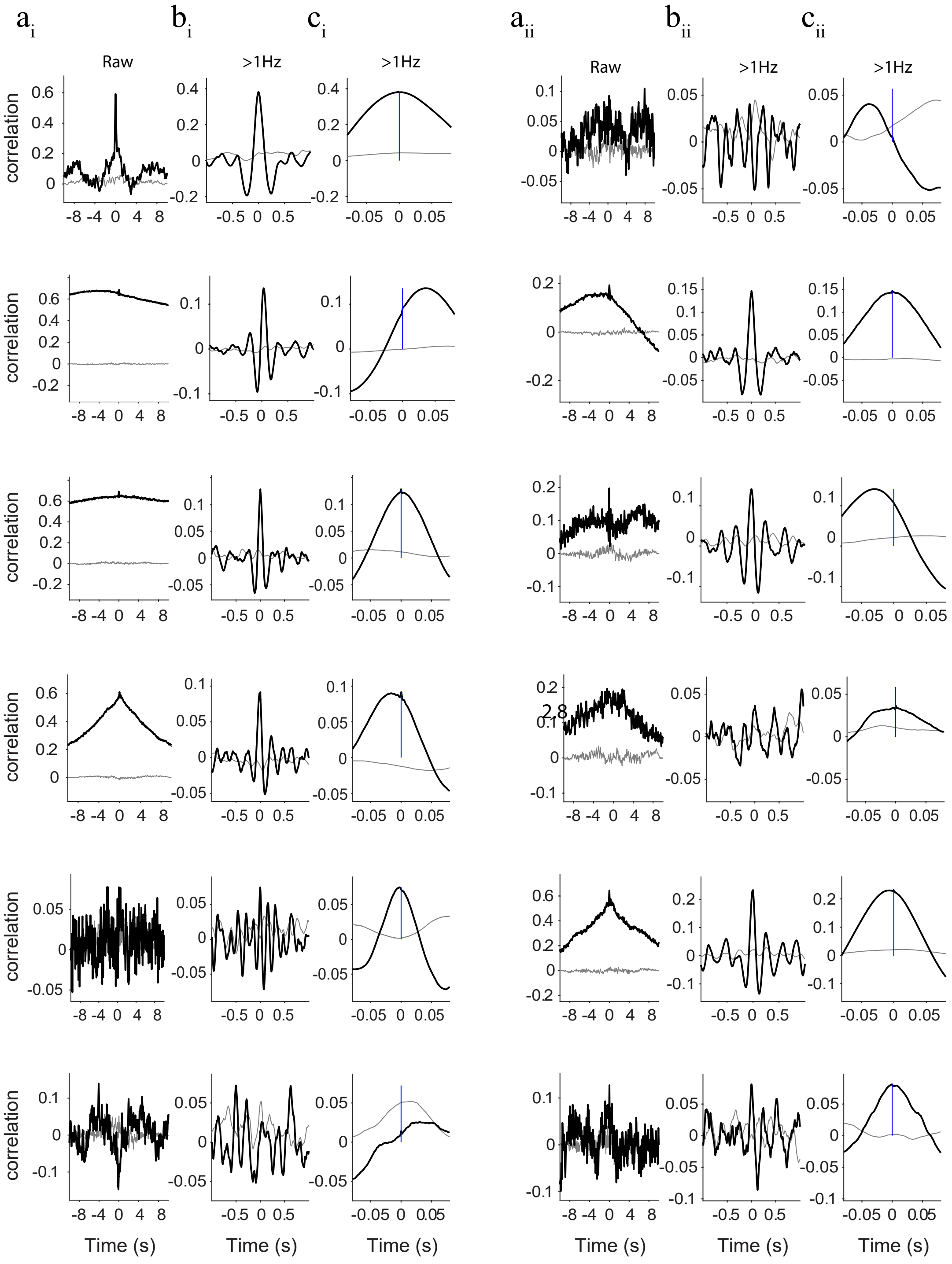

a_i,a_{ii}. Correlations between the raw membrane potentials of all the recorded pairs (black lines). **b_i,b_{ii}.** A prominent cross-correlation (black lines), higher than the shuffled data (gray lines), was found in most pairs even at the high-passed >1Hz data, indicating that part of the positive correlations between cells across hemispheres arises from fast fluctuations in membrane potential. **c_i,c_{ii}.** Same as the correlation in column b but with higher time resolution. Note that the peak sometimes is not centered at zero lag (column c, blue line marks time zero).

Supplementary Figure 6:

Interhemispheric coherence was significant up to 7Hz.

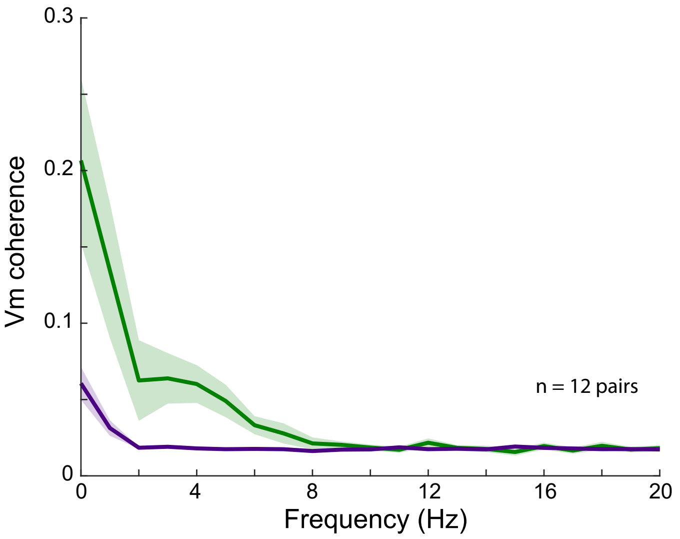

The coherence between the membrane voltages of simultaneously recorded pairs (green trace) was significantly different from shuffled data (purple trace) up to 7 Hz (WSRT. Shaded area = SEM). Shuffled-data coherence (purple line, Shaded area = SEM) was produced by calculating the coherence between Vm from one cell at time zero versus the Vm of the other cell at a random time.

Supplementary Figure 7:

The effect of light stimulation on whisking initiation in WT and GAD-ChR2 mice.

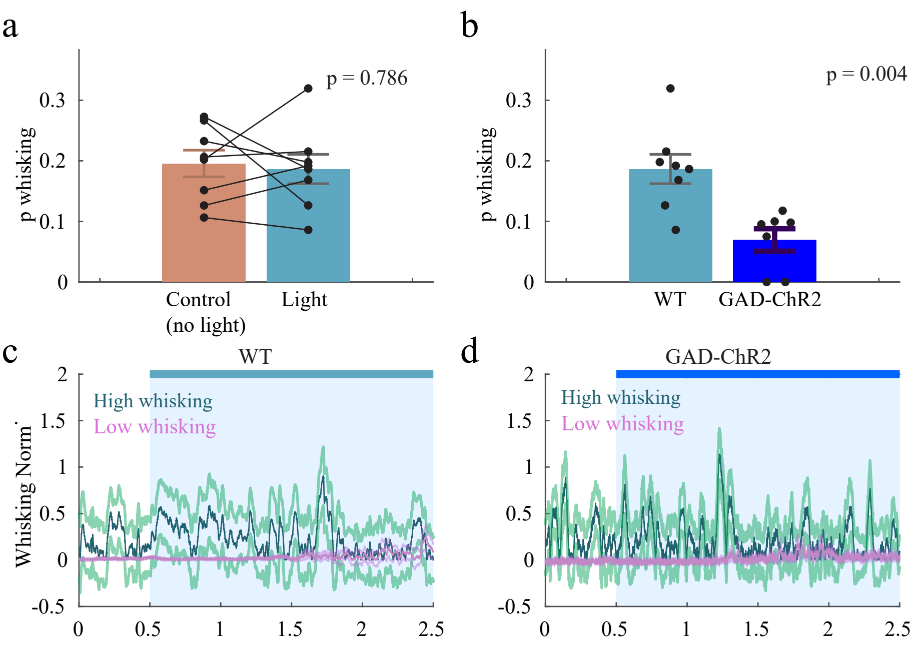

a. Probability of whisking initiation in WT mice following observing no-whisking during the 0.5 seconds before light onset (sky blue) was compared to trials in which no light was delivered (orange, n = 8 experiments in 3 animals, mean ± SEM, p = 0.786, two sided WSRT). **b.** Probability of whisking initiation following observing no-whisking in the 0.5 seconds before light onset for WT mice (sky blue) and GAD-ChR2 mice (blue, WT n = 8, GAD-ChR2 n = 7, mean ± SEM, p < 0.01, two sided WSRT). **c.** Average whisking signal in WT mice for low whisking (purple) and high whisking (teal). whisking trials were sorted similar to the analysis of the data in Figure 3. **d.** The same as in C but for the GAD-ChR2 mice. Note that for both conditions and particularly for the low-whisking case (purple line), the whisking pattern was not changed during the first 200 milliseconds after light onset. The period at which we observed a transient hyper-polarization in cells located in the hemisphere contralateral to the light-inhibited side.

Supplementary Figure 8:

Callosal lesion suppressed brain activity.

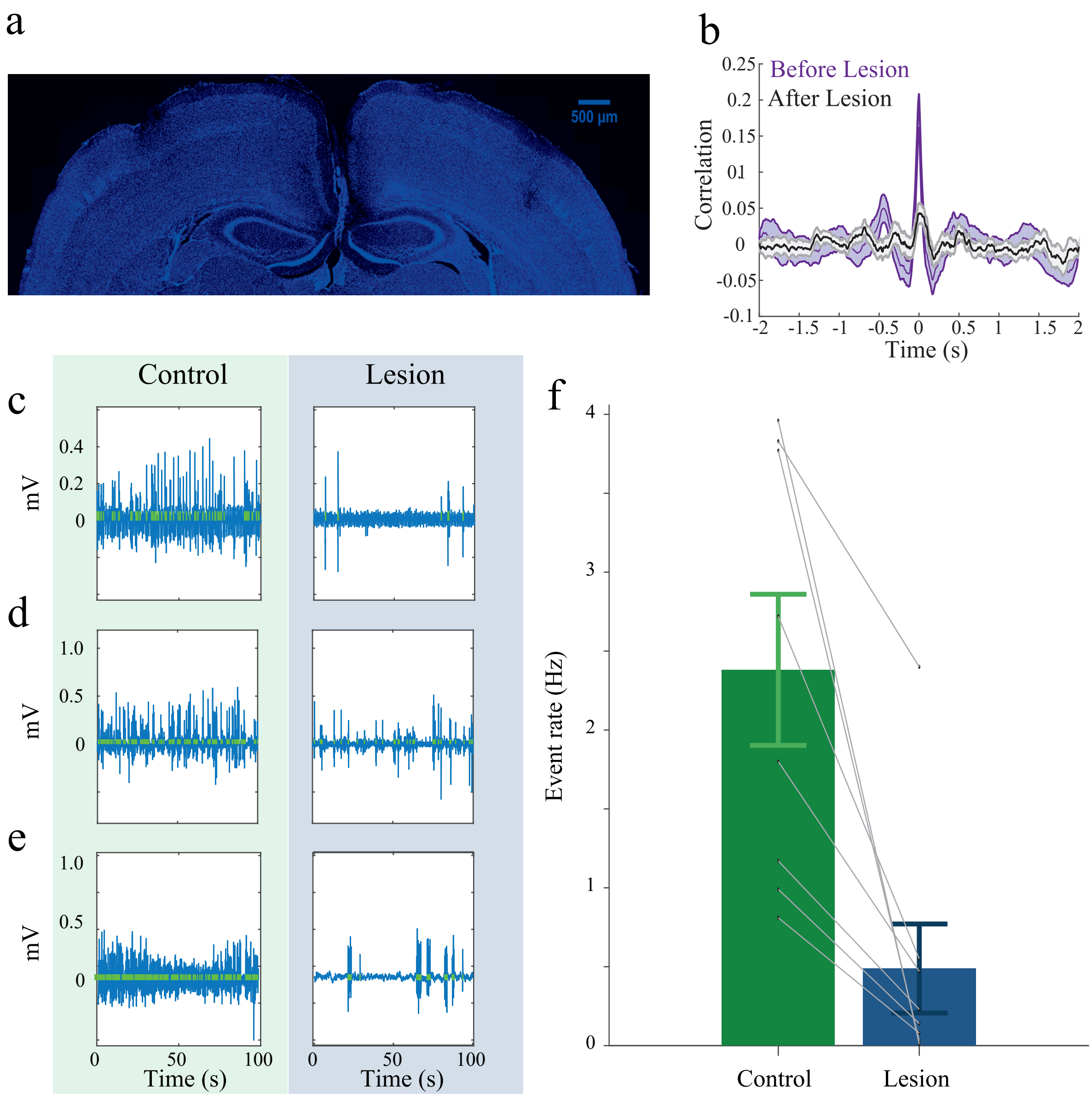

a. Histology of corpus callosum lesion (performed in 4 out of 8 animals). Scalebar 500 µm. **b.** Cross-correlation between LFP recording in the barrel cortex from the two hemispheres before (purple) and after (black) local lesion of corpus callosum between the two barrel cortices. **c, d, e.** Left panels show LFP activity in one hemisphere before suction of the corpus callosum (suction pipette was already inserted to the final location). Right panels show LFP activity following suction of the corpus callosum. Green lines mark automatically detected LFP population events. **f.** Event rate following lesion of the corpus callosum. The event rate was significantly suppressed, and ongoing brain activity was reduced (The event rate was measured by threshold-crossing events >3xSTD, n = 8 animals, mean ± SEM, $p < 0.05$, paired t-test).

Supplementary Figure 9:

The effect of CNO on the average firing rate in both hemispheres was small.

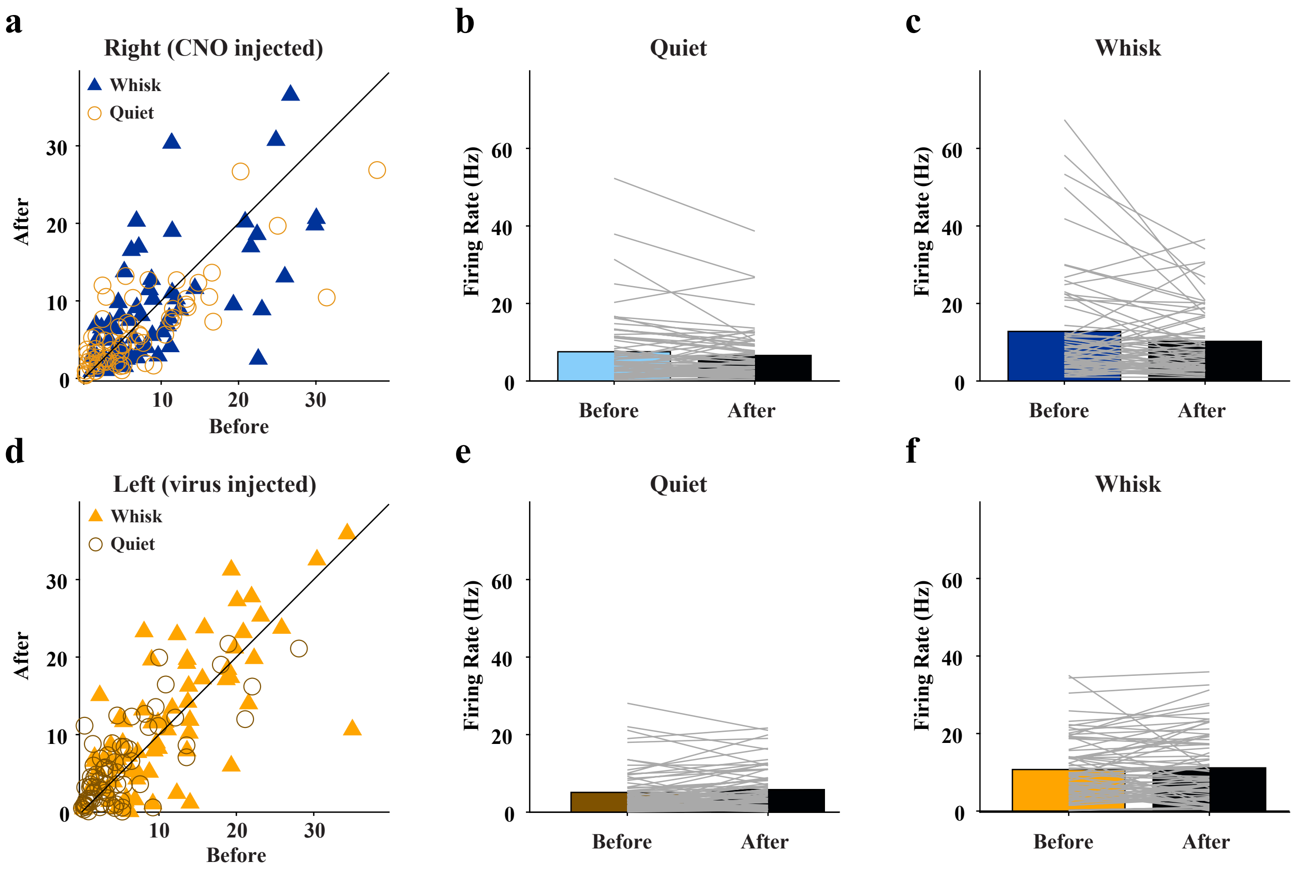

a. The mean firing rate before and after applying CNO in the right hemisphere (the hemisphere in which CNO was applied) (n=68, p>0.05, two sided WSRT). **d**. As a but in the left hemisphere (contralateral to CNO application). **b,c,e,f** Bar plot of the firing rates as shown in a and d. Note that in a and d, axes were truncated and thus a few data points are not shown, but are presented in the bar plots (p>0.05 for b,c,f, p = 0.02 for e, two sided WSRT).

Supplementary Figure 10:

The effect of CNO application on the local correlation.

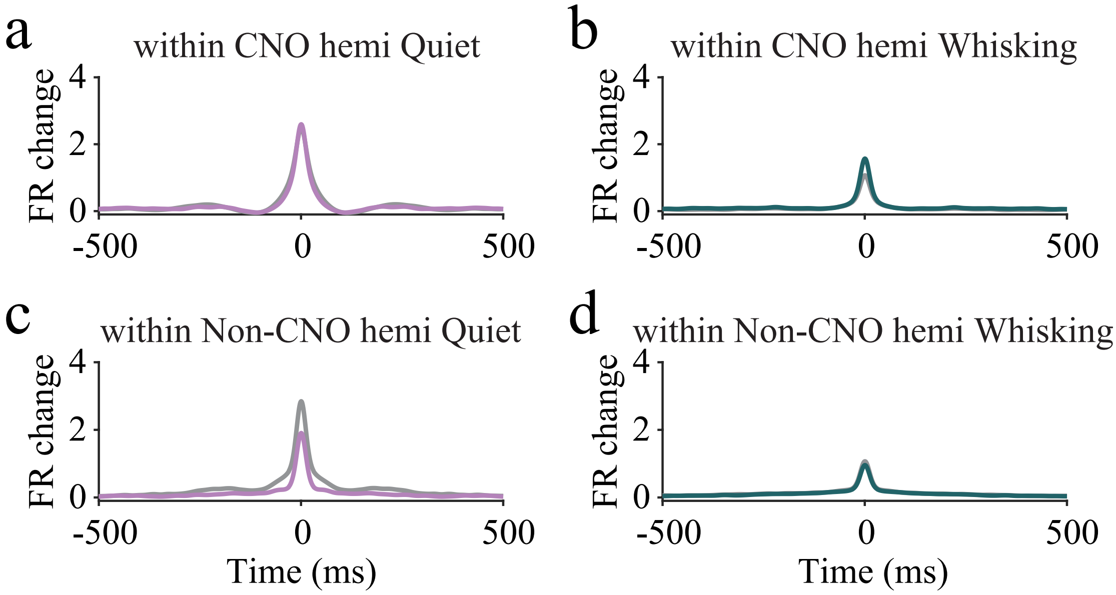

STAs were calculated before (color lines) and after the application of CNO (gray lines). a-b Local STAs for whisking and quiet epochs before application of CNO as recorded in the hemisphere in which CNO was applied (right hemisphere). c-d Local STAs for whisking and quiet epochs before applying CNO as recorded in the left hemisphere, where hM4D was expressed (Supplementary Figure 10).

Supplementary Figure 11:

Whisking pattern was not affected by the application of CNO

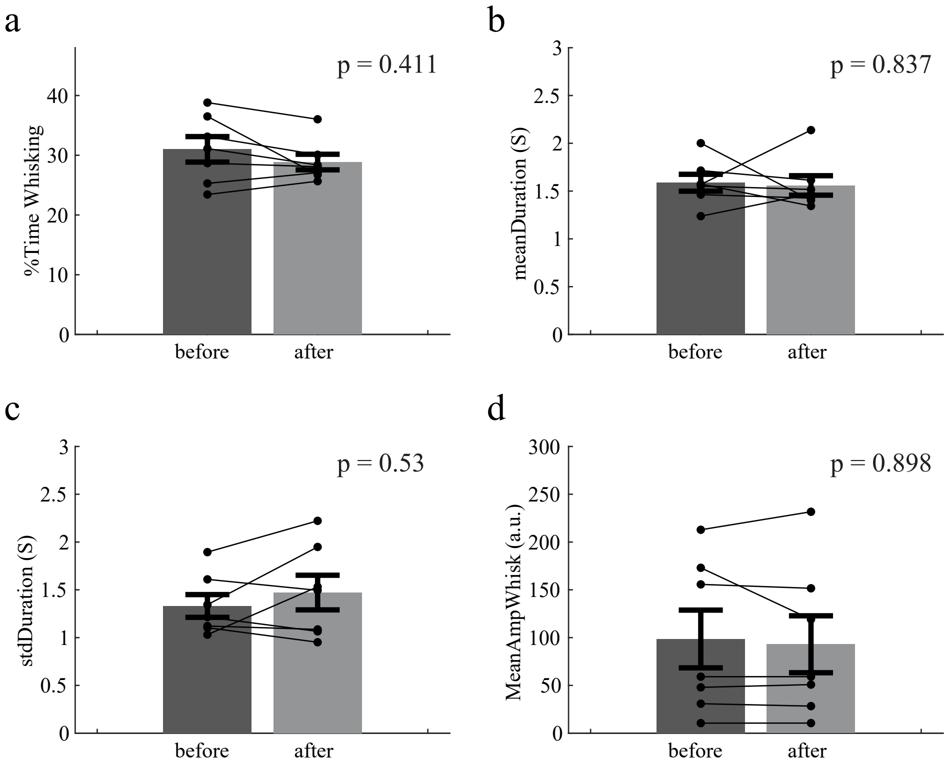

a-d. We compared the percentage of whisking time (a), mean duration of whisking epochs (b) standard deviation (c) and whisking amplitude (d) in n=7 mice. None of these properties were significantly different (two sided WSRT).

Supplementary Figure 12:

GFP imaging signals of callosal axons in awake mice are stable.

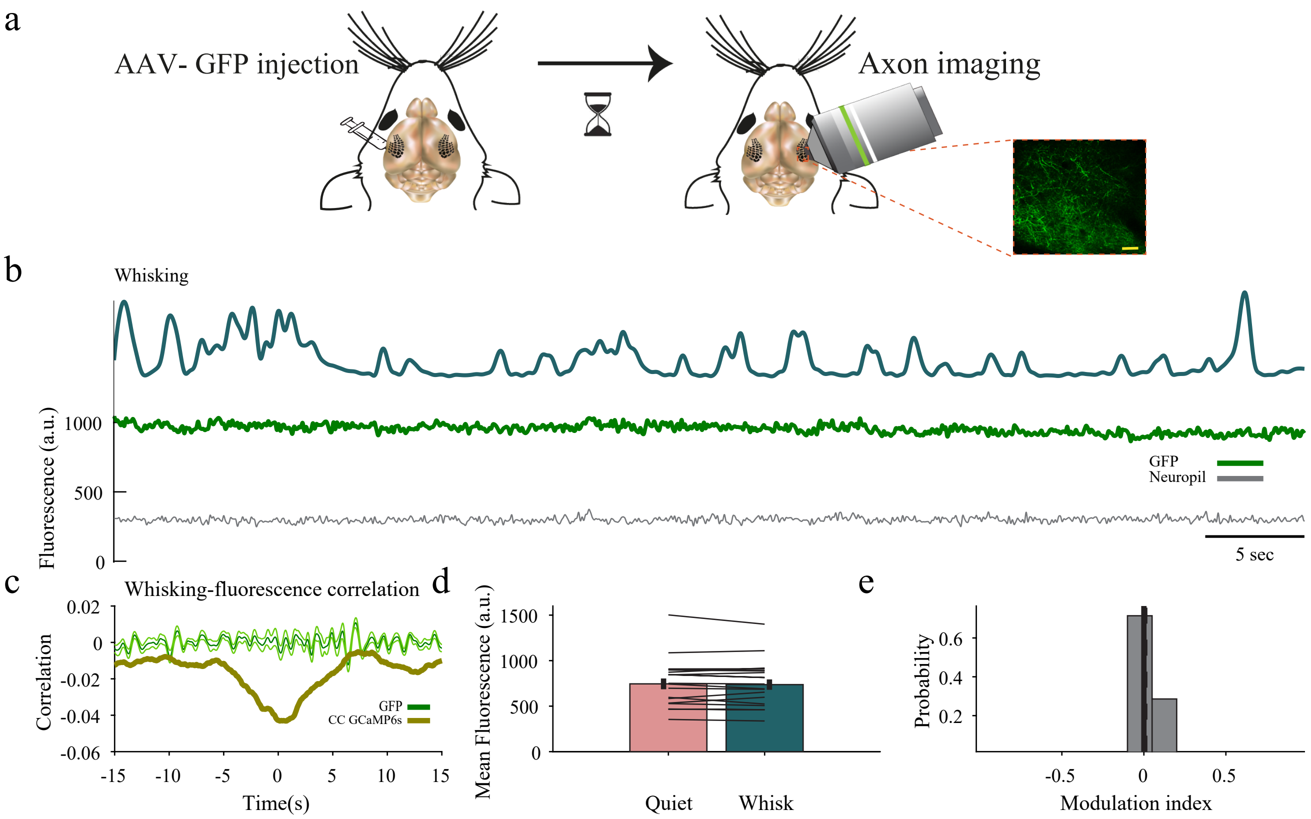

a. Schematic diagram of the injection and imaging procedures. AAV-GFP was injected in the left barrel cortex to express GFP in CaMKII cells and imaging of the contralateral hemisphere was performed three weeks later. Scalebar 50 µm. **b.** Example of simultaneous imaging of an axon (green), neuropil (gray) together with the whisking signal (teal). **c.** Cross-correlation between the callosal axonal GFP signals and whisking (teal) and between the callosal axonal GCaMP6s and whisking signals (brown). The latter is the same correlation that is presented in Figure 5c. **d.** Mean fluorescence GFP axonal signals for quiet and whisking epochs (2 animals, n = 4, 11 axons/animal, $P > 0.05$, WSRT). **e.** Histogram of the modulation index (i.e., effect of whisking on the GFP signal) for the data presented in d (dashed line marks mean MI).

Supplementary Figure 13:

Calcium imaging of CaMKII cells in upper cortical layers of awake mice shows that neuronal activity is correlated with whisking.

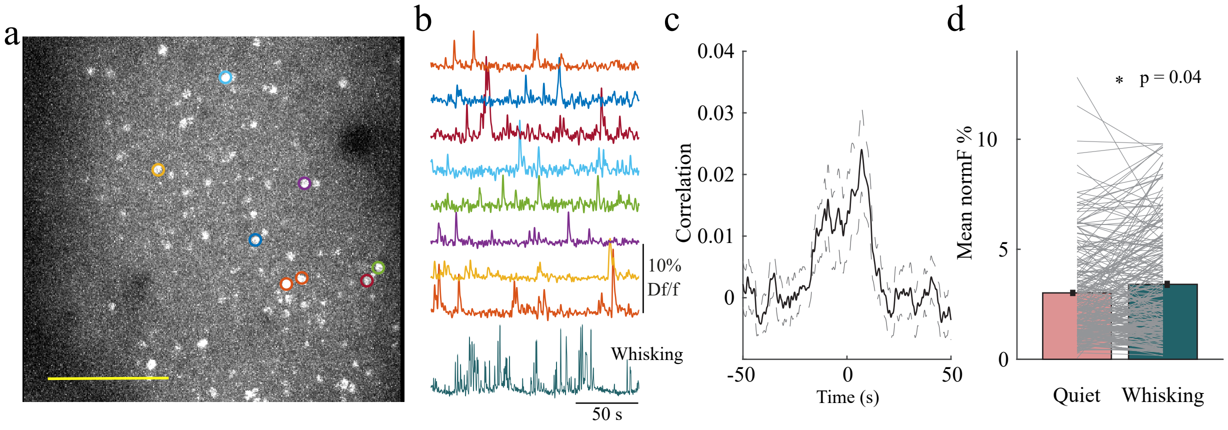

a. A picture of the imaged cortex. Scalebar 200 µm. **b.** Representative calcium signals from 8 cells and the whisking signal below. **c.** The population average of the cross-correlations between whisking and calcium signals (n = 2 mice, n = 229 cells). **d.** Mean norm calcium signals that crossed a threshold (4 times the median absolute deviation) for quiet and whisking epochs (n = 229 cells, p = 0.04, two sided WSRT).
