# Supplementary material for: Reduction of corpus callosum activity during whisking leads to interhemispheric decorrelation
Source: Nat Commun. 2021 Jul 2;12:4095. doi: 10.1038/s41467-021-24310-6 (PMC8253780; doi:10.1038/s41467-021-24310-6)
Supplement: Supplementary file 3 — Description of Additional Supplementary Files [file 41467_2021_24310_MOESM3_ESM.pdf]

Description of Additional Supplementary Files

File Name: Supplementary Movie 1
Description: A movie showing calcium imaging signals of callosal axons. The traces below depict the axonal signal (red trace obtained from the area encircled in red) simultaneously recorded with whisking activity (black).
