# Supplementary material for: Reduction of corpus callosum activity during whisking leads to interhemispheric decorrelation
Source: Nat Commun. 2021 Jul 2;12:4095. doi: 10.1038/s41467-021-24310-6 (PMC8253780; doi:10.1038/s41467-021-24310-6)
Supplement: Supplementary file 5 — Reporting Summary [file 41467_2021_24310_MOESM5_ESM.pdf]

nature research

Corresponding author(s): Ilan Lampl

Last updated by author(s): May 11, 2021

Reporting Summary

Nature Research wishes to improve the reproducibility of the work that we publish. This form provides structure for consistency and transparency in reporting. For further information on Nature Research policies, see our Editorial Policies and the Editorial Policy Checklist.

Statistics

For all statistical analyses, confirm that the following items are present in the figure legend, table legend, main text, or Methods section.

n/a	Confirmed	
☐	☒	The exact sample size (*n*) for each experimental group/condition, given as a discrete number and unit of measurement
☐	☒	A statement on whether measurements were taken from distinct samples or whether the same sample was measured repeatedly
☐	☒	The statistical test(s) used AND whether they are one- or two-sided<br>*Only common tests should be described solely by name; describe more complex techniques in the Methods section.*
☒	☐	A description of all covariates tested
☐	☒	A description of any assumptions or corrections, such as tests of normality and adjustment for multiple comparisons
☐	☒	A full description of the statistical parameters including central tendency (e.g. means) or other basic estimates (e.g. regression coefficient) AND variation (e.g. standard deviation) or associated estimates of uncertainty (e.g. confidence intervals)
☐	☒	For null hypothesis testing, the test statistic (e.g. *F*, *t*, *r*) with confidence intervals, effect sizes, degrees of freedom and *P* value noted<br>*Give P values as exact values whenever suitable.*
☒	☐	For Bayesian analysis, information on the choice of priors and Markov chain Monte Carlo settings
☒	☐	For hierarchical and complex designs, identification of the appropriate level for tests and full reporting of outcomes
☐	☒	Estimates of effect sizes (e.g. Cohen's *d*, Pearson's *r*), indicating how they were calculated

Our web collection on statistics for biologists contains articles on many of the points above.

Software and code

Policy information about availability of computer code

Data collection: (1) Custom-made programs in LabVIEW 2018 (electrophysiology recording), (2) Open Ephys (version 0.5.2.2), (extracellular recordings), and (3) PrairieView 5.4 (2P imaging).

Data analysis: (1) Custom-written code in MATLAB 2019b and 2018a (analysis of electophysiology and 2P imaging), (2) Suite2P (preprocessing of 2P imaging) , (3) LabVIEW 2018 (preprocessing of 2P imaging and behavior), (4) wave_clus toolbox for MATLAB (spike sorting).

For manuscripts utilizing custom algorithms or software that are central to the research but not yet described in published literature, software must be made available to editors and reviewers. We strongly encourage code deposition in a community repository (e.g. GitHub). See the Nature Research guidelines for submitting code & software for further information.

Data

Policy information about availability of data

All manuscripts must include a data availability statement. This statement should provide the following information, where applicable:

- Accession codes, unique identifiers, or web links for publicly available datasets
- A list of figures that have associated raw data
- A description of any restrictions on data availability

(1) Source Data of the figures are available in a zipped file containing Excel files. (2) Processed data are available in a repository link as shown in the manuscript. (3) Raw data are available from the corresponding author upon reasonable request.

Field-specific reporting

Please select the one below that is the best fit for your research. If you are not sure, read the appropriate sections before making your selection.

☒ Life sciences ☐ Behavioural & social sciences ☐ Ecological, evolutionary & environmental sciences

For a reference copy of the document with all sections, see nature.com/documents/nr-reporting-summary-flat.pdf

Life sciences study design

All studies must disclose on these points even when the disclosure is negative.

Sample size	No sample-size calculations were performed. Sample size (i.e., number of mice and recorded or imaged cells) was determined according to the standards in the field for cellular electrophysiology and imaging (Cohen-Kashi Malina et al. 2016 doi: 10.1038/ncomms12740; Reimer et al. 2016 DOI: 10.1038/ncomms13289; Arroyo et al. 2018 DOI: 10.1016/j.neuron.2018.08.008; Nowak et al. 1995 DOI: 10.1152/jn.1995.74.6.2379);
Data exclusions	Exclusions in intracellular physiology were based on resting potential and spike amplitude, namely if mean resting potential was > -50 mV or spike amplitude was less than 40 mV, either of which is indicative of cell damage or bad sealing of the patch pipette to the membrane. Exclusions for imaged animals were based on a lack of GCaMP expression. No other exclusions were made.
Replication	Replication was done across trials, recorded units/cells/cell-pairs/axons and animals. The exact number of repetitions (cells or animals) are indicated in figures or figure legends. The number of trials per individual unit/cell/cell-pair/axon is indicated along with the number of such recordings as well as the number of animals.
Randomization	Mice of a given strain (for each experiment) were randomly taken from the breeding house and assigned to the different manipulation groups. Randomization was irrelevant to data collection and manipulation due to lack of trial structure except for optogenetic stimulation trials which were randomized with no-light trials.
Blinding	Blinding was not relevant to this study because during the electrophysiological recordings, imaging and manipulations no information about the meaning of the observed activity could be inferred and thus no experimenter bias could be introduced. Offline analysis was performed using automated computer routines which similarly could not be affected by experimenter bias.

Reporting for specific materials, systems and methods

We require information from authors about some types of materials, experimental systems and methods used in many studies. Here, indicate whether each material, system or method listed is relevant to your study. If you are not sure if a list item applies to your research, read the appropriate section before selecting a response.

Materials & experimental systems

n/a	Involved in the study	
☒	☐	Antibodies
☒	☐	Eukaryotic cell lines
☒	☐	Palaeontology and archaeology
☐	☒	Animals and other organisms
☒	☐	Human research participants
☒	☐	Clinical data
☒	☐	Dual use research of concern

Methods

n/a	Involved in the study	
☒	☐	ChIP-seq
☒	☐	Flow cytometry
☒	☐	MRI-based neuroimaging

Animals and other organisms

Policy information about studies involving animals; ARRIVE guidelines recommended for reporting animal research

Laboratory animals	Mice of either sex, 7-15 weeks. 1) GAD2-IRES-Cre mice (JAX, stock #010802) crossed with Ai32 (Stock #012569). 2) C57BL/6 . Mice were housed under 12-h light/dark cycle and 21 degrees C, 33% humidity.
Wild animals	This study did not involved wild animals.
Field-collected samples	This study did not involved samples collected from field.
Ethics oversight	All experiments were conducted according to the Weizmann Institute Institutional Animal Care and Use Committee.

Note that full information on the approval of the study protocol must also be provided in the manuscript.
